# Impact of the COVID-19 Pandemic on Mental Well-Being. A Nationwide Online Survey Covering Three Pandemic Waves in Poland

**DOI:** 10.3389/fpsyt.2021.804123

**Published:** 2021-12-17

**Authors:** Mateusz Babicki, Krzysztof Kowalski, Bogna Bogudzińska, Agnieszka Mastalerz-Migas

**Affiliations:** ^1^Department of Family Medicine, Wroclaw Medical University, Wrocław, Poland; ^2^Department and Clinic of Psychiatry, Wroclaw Medical University, Wrocław, Poland; ^3^Students' Scientific Group at the Faculty of Psychiatry, Wroclaw Medical University, Wrocław, Poland

**Keywords:** COVID-19, anxiety, depression, quality of life, mental health

## Abstract

The COVID-19 pandemic has a significant impact on human life. This study aims to assess the prevalence of depressive and anxiety symptoms, and the assessment of the quality of life in different stages of the COVID-19 pandemic based on an online nationwide survey. The study was based on a voluntary, anonymous, and authors' own questionnaire. The first section assesses sociodemographic status. Then, standardized psychometric tools were used such as the Beck Depression Inventory (BDI), the Generalized Anxiety Disorder Assessment (GAD-7), and the Manchester Short Assessment of Quality of Life (MANSA). The study was conducted in three stages corresponding to the waves of the COVID-19 pandemic in Poland. The survey involved 5,790 respondents; 2,457, 1,626, and 1,707 for the first, second, and third pandemic wave, respectively. It was found that anxiety and depressive symptoms increased as the pandemic progressed. There was no significant effect on the subjective quality-of-life assessment. Moreover, there was a gradual decrease in anxiety about being infected with COVID-19 as well as reduced adherence to the Minister of Health's recommendations. As the COVID-19 pandemic progressed, depressive and anxiety symptoms increased among Poles. Women, singles, and people with prior psychiatric treatment are more likely to develop the aforementioned symptoms.

## Introduction

A few months after the first viral pneumonia of SARS-CoV-2 etiology was diagnosed and due to the rapid spread of SARS-CoV-2 around the world, the WHO declared a pandemic (1–3). Although safe and effective vaccines against COVID-19 were invented, their worldwide availability and, in some countries, the degree of their public acceptance is too low to stop the pandemic at this stage (4). Therefore, keeping a safe distance from others is still part of the fight against the pandemic. As the pandemic continued, the Polish government—as many other governments around the world—made decisions to limit, and sometimes even completely close many sectors of the economy. Furthermore, schools, and kindergartens were closed (5). The introduced restrictions evoked many emotions and controversy about their validity and effectiveness, while significantly changing and complicating daily professional, and social functioning (5, 6). Prolonged feelings of fear and uncertainty about the future as well as separation from loved ones have contributed to a significant increase in the prevalence of anxiety, and depressive disorders in the population (7). Scientific reports clearly indicate that the increase of these symptoms is concurrent with the pandemic progression and they are more prevalent in women and singles (8). Moreover, these scientific reports imply that the psychological stress associated with COVID-19 is not a short-term condition and it may contribute to chronic mental health disorders that are similar to those described in post-traumatic stress disorder (8). UK observations revealed that the rise of the third wave of the pandemic led to an increase in the incidence of suicidal thoughts—especially in young people. People with lower socioeconomic status and prior psychiatric treatment also suffer from poorer mental health. Losing your job and the resulting worse financial situation lead to an increased sense of helplessness (9). Although effective vaccines are available in developed countries, it is still uncertain when the pandemic will end and the associated problems will disappear (10). The prolonged and variable course of the pandemic, successive pandemic waves and emergence of new SARS-CoV-2 variants point to a high likelihood of further public mood volatility, as well as an increase in mental disorders associated with chronic stress (8). This study aims to assess the prevalence of depressive and anxiety symptoms, and the subjective assessment of the quality of life in different stages of the COVID-19 pandemic based on an online nationwide survey. Based on previous knowledge, the following research hypotheses were posed: (1) The ongoing epidemic situation has an negative impact on mental health. (2) Women and singles have poorer mental health. (3) Economic instability significantly worsens the mental condition.

## Materials and Methods

### Methodology

The study was based on the authors' own questionnaire distributed online through a social networking site. Participation in the study was fully anonymous and voluntary. The questionnaire was addressed to all persons living in Poland, aged 18 or older, with access to the Internet. Before the respondents took part in the study, they were informed about the nature of the study, its methodology and objectives. Informed consent was then obtained from those willing to participate. The participants were free to withdraw from the study at any stage without giving any reason. The study consisted of three consecutive stages of survey distribution. The first stage covered the early days of the pandemic in Poland—from 17 to 26 April 2020, i.e., less than a month after the first confirmed case of COVID-19 in Poland. That was the period when 263 to 460 cases of COVID-19 were diagnosed in Poland per day and the number of deaths fluctuated between 18 and 40 (11). To inhibit the spread of SARS-CoV-2, the Polish government decided to implement several restrictions that covered many areas of daily life, including closing schools, shops except for grocery shops, theaters, cinemas, swimming pools, gyms, restaurants (only take-out food), hairdressing salons, and hotels (12). The second stage of the study was conducted during the period of the next in-crease in SARS-CoV-2 cases in Poland, referred to as the “second wave of the pandemic”; the questionnaire was distributed from 1 to 30 December 2020. During that time, the number of COVID-19 cases fluctuated between 2,921–14,835 cases and between 29–620 deaths per day (11). Restrictions used in the first wave of the pandemic were reimplemented with the exception of the closure of shopping malls, hairdressing salons, and beauty salons (13). The third stage of the study covered the period of the highest incidence and death rates in Poland due to COVID-19. Data were collected from 20 March 2021 to 30 April 2021 when the daily incidence rate ranged from 6,802 to 35,246 COVID-19 cases, with daily deaths ranging from 428 to 954 (11). Faced with dramatic rates, the Polish government decided to implement a series of restrictions that were much more restrictive than those implemented in previous stages. Those restrictions included the closure of shopping malls, DIY shops, excluding i.e., grocery shops, pharmacies, beauty supply shops, newsagent's shops, bookshops. Hairdressing salons and beauty salons, sports facilities, including gyms and fitness centers, were closed, and only professional sports activities could take place—without any visitors present. Schools, nurseries and kindergartens were closed—the last two remaining open only for children of healthcare professionals. Art galleries, museums, and theaters were also closed. There was a strong emphasis on doing remote work wherever possible (14).

The study was approved by the Bioethics Committee of the Wroclaw Medical University and was conducted in accordance with the Declaration of Helsinki.

The questionnaire designed for this study and prepared by the authors consisted of several sections. The first section included the sociodemographic status of the respondents including age, sex, place of residence, level of education, marital status, and being a healthcare professional. Moreover, past medical history of mental disorders and COVID-19 infection, as well as its suspicion, was assessed. To assess the level of anxiety about contracting COVID-19 infection, the authors used their own set of questions based on a 10-point Likert scale (1—no anxiety, 10—extreme anxiety) concerning both subjective anxiety about being infected with SARS-CoV-2 and the level of anxiety about neighbors in quarantine or neighbors being infected with COVID-19. The subjective assessment of adherence to the Ministry of Health recommendations regarding COVID-19 prevention.

Another section consisted of standardized psychometric tools such as the Beck Depression Inventory (BDI), the Generalized Anxiety Disorder Assessment (GAD-7), and the Manchester Short Assessment of Quality of Life (MANSA).

(1) *The Beck Depression Inventory (BDI)* is one of the most commonly used psycho-metric tools consisting of 21 questions, in which the respondent makes a subjective assessment of the severity of a particular mental state on a scale from 0 to 3. To interpret the results, the following cut-off points were applied: 0–11—no depression, 12–26 points—mild depression; 27–49—moderate depression; 50–63—severe depression (15).
(2) *The Generalized Anxiety Disorder Assessment (GAD-7)* is a 7-item tool based on a 4-point Likert scale. Respondents assess the frequency of occurrence of a particular mental state during the last 14 days (0—not at all, 1—a few days, 2—more than half the time, 3—almost always). The maximum number of available points to score was 21. The analysis of the tool is based on 3 cut-off points: 5, 10, and 15 points that indicate the presence of mild, moderate, and severe anxiety, respectively. A score of at least 10 points indicated a high probability of generalized anxiety disorder (16).
(3) *The Manchester Short Assessment of Quality of Life (MANSA)* is a tool derived from the Lancashire Quality of Life Profile (LQLP) while keeping its parametric values. It is used for the subjective assessment of the quality of life by rating 16 aspects of life on a 7-point Likert scale (1—could not be worse, 7—could not be better). The analysis of the tool is based on the total score—the higher the score, the higher the quality of life. The analysis of the tool can also be done at the level of individual questions (17, 18).

### Statistical Analysis

The statistical analysis was performed using R 4.1.0 and Statistica 14.0.0.15.

Variables were of qualitative and quantitative nature. Basic descriptive statistics methods were used for the quantitative variables. Furthermore, the Lilliefors test was used for assessing the normality of the distribution and the Brown-Forsythe test was used for assessing the variance. When the assumption of equality of variance across subgroups for quantitative variables was not met, the Welch ANOVA was performed. Subsequently, the *post-hoc* analysis was performed using the Games-Howell test. The Pearson's chi-squared test with Bonferroni correction was used for comparing qualitative variables. Linear models were used for the assessment of the relationship be-tween sociodemographic variables and final scale scores. The Spearman's correlation analysis was used for assessing the correlation between different scales.

Statistical significance level was established at *p* < 0.05 for each case.

## Results

### Characteristics of the Study Group

A detailed profile of the study group is shown in Table 1. The study included 5,790 participants. At each stage, the overwhelming majority of respondents were women and those living in a city with a population of over 250,000. In the first, second and third stage of the study, the mean age of the respondents was 32.2 ± 10.72, 24.6 ± 7.06, and 27.83 ± 9.55 years, respectively. As the study progressed, the percentage of both individuals and their relatives who were COVID-19 convalescents increased. Also, as the pandemic progressed, there was a downward trend in COVID-19-related information retrieval and daily tracking of death and hospitalization statistics.

**Table 1:** Characteristics of the study group by study stage.

**Variable**		**Stage 1** (***n*** = 2,467) M ± SD^*^/***N*** **(%)**	**Stage 2** (***n*** **= 1,627)** M ± SD^*^/***N*** **(%)**	**Stage 3** (***n*** = 1,696) M ± SD^*^/***N*** **(%)**	***p***
Age^*^		32.2 ± 10.72	24.6 ± 7.06	27.83 ± 9.55	<0.001
Sex	Female	2,037 (82.5)	1,295 (79.6)	1,394 (82.2)	0.0229
	Male	430 (17.5)	332 (20.4)	302 (17.8)	
Place of residence	Rural area	461 (18.7)	287 (17.6)	326 (19.2)	0.0749
	Town of up to 50,000 inhabitants	377 (15.3)	233 (14.4)	268 (15.8)	
	City of 50,000–250,000 inhabitants	449 (18.2)	303 (18.6)	353 (20.8)	
	City of over 250,000 inhabitants	1,180 (47.8)	804 (49.4)	744 (44.2)	
Level of education	Higher (university degree)	1,481 (60.0)	513 (31.5)	654 (38.6)	<0.001
	Incomplete higher	514 (20.8)	646 (39.6)	543 (32.1)	
	Secondary	429 (17.4)	437 (26.9)	445 (26.4)	
	Vocational	26 (1.0)	8 (0.5)	9 (0.5)	
	Lower secondary	13 (0.6)	19 (1.2)	24 (1.4)	
	Primary	4 (0.2)	4 (0.3)	9 (0.5)	
Marital status	Married	867 (35.1)	163 (10.0)	323 (19.0)	<0.001
	Partnership	556 (22.6)	446 (27.5)	475 (28.0)	
	Widowed	30 (1.2)	7 (0.4)	14 (0.8)	
	Divorced	108 (4.4)	25 (1.5)	50 (3.0)	
	Single	905 (36.7)	986 (60.6)	834 (49.2)	
Healthcare professional	Yes	632 (25.6)	203 (12.5)	245 (14.5)	<0.001
	No	1,835 (74.4)	1,424 (87.5)	1,451 (85.5)	
Prior psychiatric treatment	Yes	516 (20.9)	333 (20.5)	340 (20.1)	0.7899
	No	1,951 (19.1)	1,294 (79.5)	1,356 (79.9)	
Psychiatric drug treatment	Yes	443 (18.0)	268 (16.5)	283 (16.7)	0.3846
	No	2,024 (82.0)	1,359 (83.5)	1,413 (83.3)	
COVID-19 infection suspected	Yes	78 (3.2)	323 (19.9)	352 (20.8)	<0.001
	No	2,389 (96.8)	1,304 (80.1)	1,344 (79.2)	
Forced quarantine	Yes, I am under home isolation	23 (0.9)	29 (1.8)	31 (1.8)	<0.001
	Yes, I was under home isolation	59 (2.4)	243 (14.9)	314 (18.5)	
	No	2,385 (95.7)	1,355 (82.3)	1,351 (79.7)	
Diagnosed with COVID-19	In the course of the disease	189 (7.9)	33 (2.0)	39 (2.3)	<0.001
	Yes, I was infected with COVID-19 in the past	143 (6.0)	248 (15.2)	298 (17.6)	
	No	2,056 (86.1)	1,346 (82.8)	1,359 (80.1)	
COVID-19 diagnosed in loved ones	Yes	117 (4.7)	1,036 (63.7)	1,122 (66.2)	<0.001
	No	2,350 (95.3)	591 (36.3)	574 (33.8)	
Information retrieval	Yes	1,530 (62.0)	776 (47.7)	767 (45.22)	<0.001
	No	937 (38.0)	851 (52.3)	929 (54.8)	
Tracking statistics on COVID-19	Yes	1,562 (63.3)	781 (48.0)	710 (41.9)	<0.001
	No	905 (36.7)	846 (52.0)	986 (58.1)	
Loss of income opportunities	Yes	610 (24.7)	340 (20.9)	359 (21.2)	0.0039
	No	1,857 (75.3)	1,287 (79.1)	1,337 (78.8)	

### R Analysis of BDI, GAD-7, and MANSA for Each Wave of the COVID-19 Pandemic

The mean GAD-7 score increases in successive waves of the COVID-19 pandemic; however, there are no significant differences between the first and second wave or between the second and third wave. There is a statistically significant difference between the first and third wave (*p* = 0.001). The mean BDI score increases in successive waves of the COVID-19 pandemic; however, there is a significantly greater increase between the first and second wave than between the second and third wave. Stated differently, respondents revealed a lower sense of depression and anxiety in the first wave than in successive waves. The mean MANSA score does not have either increasing or decreasing trend. The analysis of individual questions included in the MANSA scale showed that as the COVID-19 pandemic continued, a significant decrease in satisfaction with one's mental condition, financial condition, and one's additional activities (hobbies) was observed. However, some aspects have improved, mainly relationships with family and roommates. A detailed breakdown of the MANSA scores is presented in Supplementary Table 1.

The results are summarized in Tables 2, 3.

**Table 2:** Comparison of BDI, GAD-7, and MANSA scores according to different stages of the study.

**BDI Wave**	**Wave**	**Difference in means**	**Lower end of the range for differences confidence intervals**	**Upper end of the range for differences**	***P*** ^*^
1	2	0.0971677	0.0617	0.133	**0.000**
1	3	0.1384415	0.102	0.175	**0.000**
2	3	0.0412738	0.0002	0.082	**0.048**
**GAD-7**					
1	2	0.0559536	−0.009	0.121	0.109
1	3	0.1006945	0.034	0.166	**<0.001**
2	3	0.0447409	−0.026	0.116	0.306
**MANSA**					
1	2	0.0574767	−0.002	0.117	0.060
1	3	−0.0271350	−0.087	0.033	0.536
2	3	−0.0846117	−0.150	−0.019	**0.007**

* *(Welch's) ANOVA univariate. Significant effects (<0.05) are marked in bold*.

**Table 3:** Comparison of individual COVID-19 pandemic waves between BDI and GAD-7 interpretations.

**Stage of the study**	**Stage of the study**	***p*** ^*^
**BDI**		
First wave	Second wave	**<0.0001**
First wave	Third wave	**<0.0001**
Second wave	Third wave	0.125
**GAD-7**		
First wave	Second wave	0.376
First wave	Third wave	**0.001**
Second wave	Third wave	0.812

* *Pearson's chi-squared test with Bonferroni correction. Significant effects (<0.05) are marked in bold*.

The BDI interpretation revealed a statistically significant difference between individual pandemic waves (*p* < 0.0001), as did GAD-7 (*p* = 0.004). The comparison of individual waves of the COVID-19 pandemic revealed a significant difference in terms of the distribution of BDI interpretations between the first and second wave and between the first and third wave. GAD-7 revealed the difference between COVID-19 pandemic waves only when comparing the first wave and third wave.

**Figure 1:**
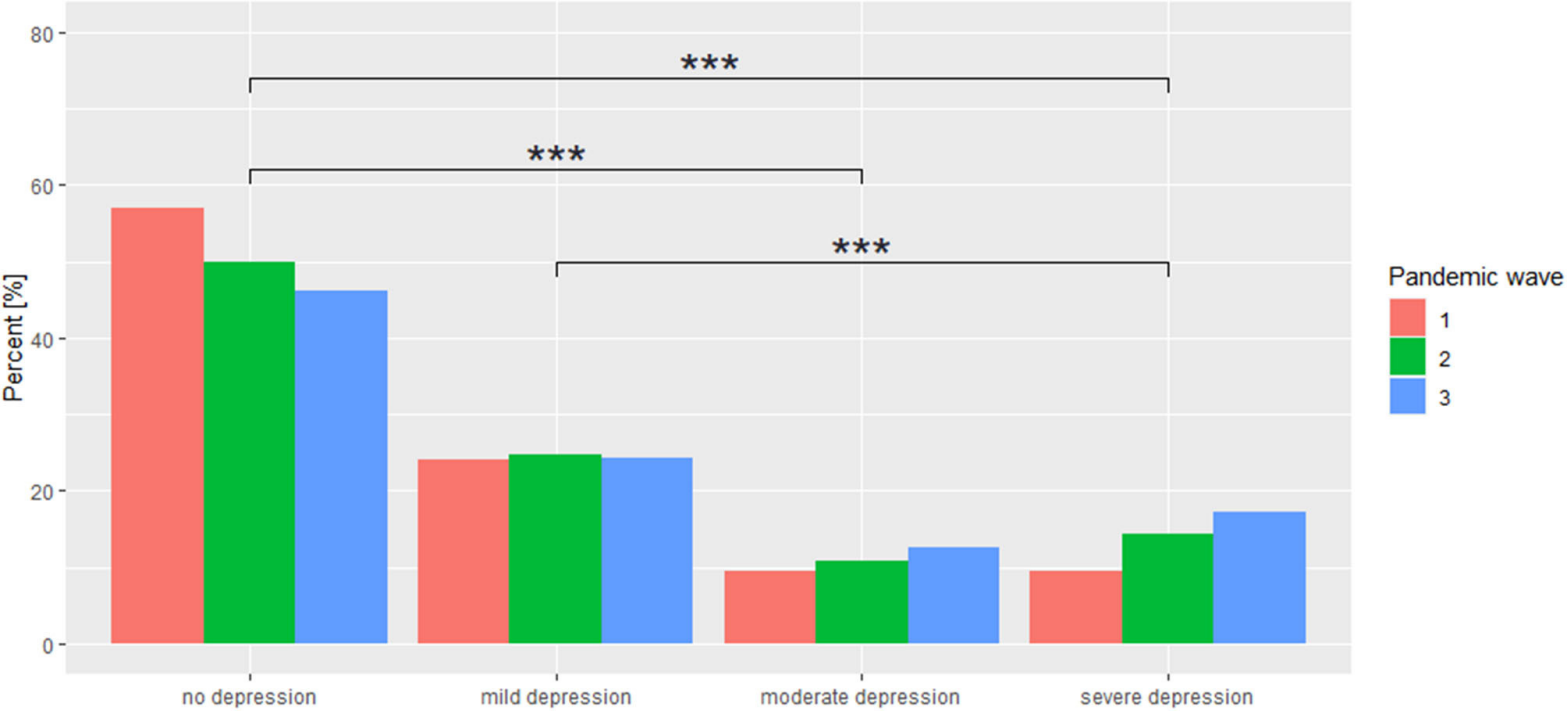
Distribution of BDI interpretations across individual COVID-19 pandemic waves. ^***^p < 0.001.

As the pandemic continues, there is an increasing trend in the percentage of individuals whose BDI score indicates the presence of depressive disorders. Moreover, there is an increase in the percentage of individuals with moderate and severe depression. There is no statistically significant difference between adjacent interpretations (no depression—mild depression, mild depression—moderate depression, moderate depression—severe depression); however, there is a statistically significant difference for the pairs such as no depression—moderate depression, no depression—severe depression, and mild depression—severe depression. This may be indicative of systematically progressive unidirectional changes. The results are shown in Table 4. The exact distribution of BDI interpretations is shown in Figure 1.

**Table 4:** Pairwise comparison of BDI interpretations between individual waves of the COVID-19 pandemic.

**Interpretation**		***P*** ^*^
No depression	Mild depression	0.0874
No depression	Moderate depression	**0.0001**
No depression	Severe depression	**<0.0001**
Mild depression	Moderate depression	0.3272
Mild depression	Severe depression	**<0.0001**
Moderate depression	Severe depression	0.1443

* *Pearson's chi-squared test with Bonferroni correction. Significant effects (<0.05) are marked in bold*.

**Figure 2:**
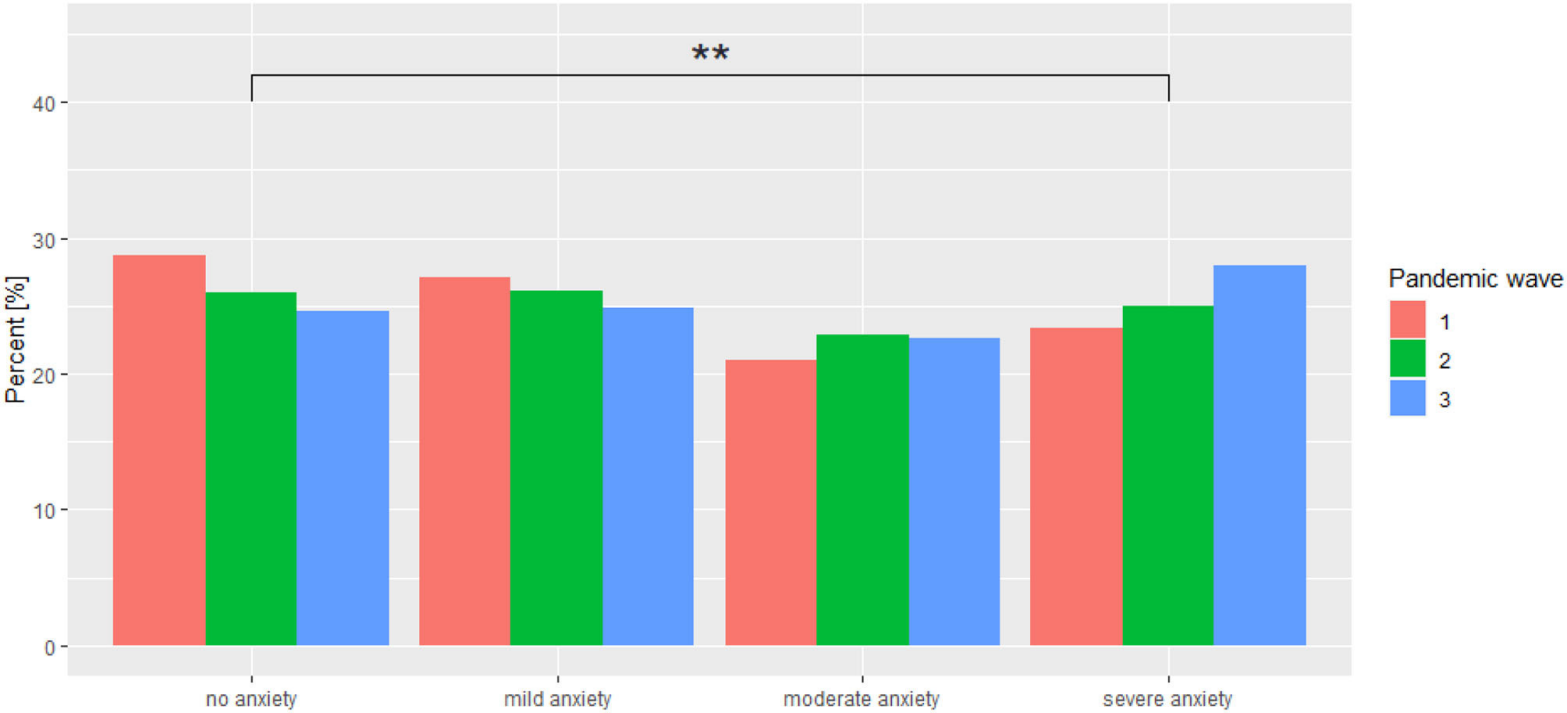
Distribution of GAD-7 interpretations across individual COVID-19 pandemic waves. ^**^p < 0.01.

There is no statistically significant difference between adjacent interpretations (no anxiety—mild anxiety, mild anxiety—moderate anxiety, moderate anxiety—severe anxiety), as well as between pairs such as no anxiety—moderate anxiety, mild anxiety—severe anxiety, while there is a statistically significant difference in terms of the distribution of COVID-19 pandemic waves for the pair no anxiety—severe anxiety. The results are shown in Table 5. Extreme GAD-7 scores changed significantly during the pandemic (Figure 2).

**Table 5:** Pairwise comparison of GAD-7 interpretations between individual waves of the COVID-19 pandemic.

**Interpretation**		***P*** ^*^
No anxiety	Mild anxiety	1
No anxiety	Moderate anxiety	0.129
No anxiety	Severe anxiety	**0.003**
Mild anxiety	Moderate anxiety	1
Mild anxiety	Severe anxiety	0.058
Moderate anxiety	Severe anxiety	1

* *Pearson's chi-squared test with Bonferroni correction. Significant effects (<0.05) are marked in bold*.

### Anxiety About Being Infected With COVID-19 and Adherence to the Ministry of Health Recommendations Regarding COVID-19 Prevention

**Figure 3:**
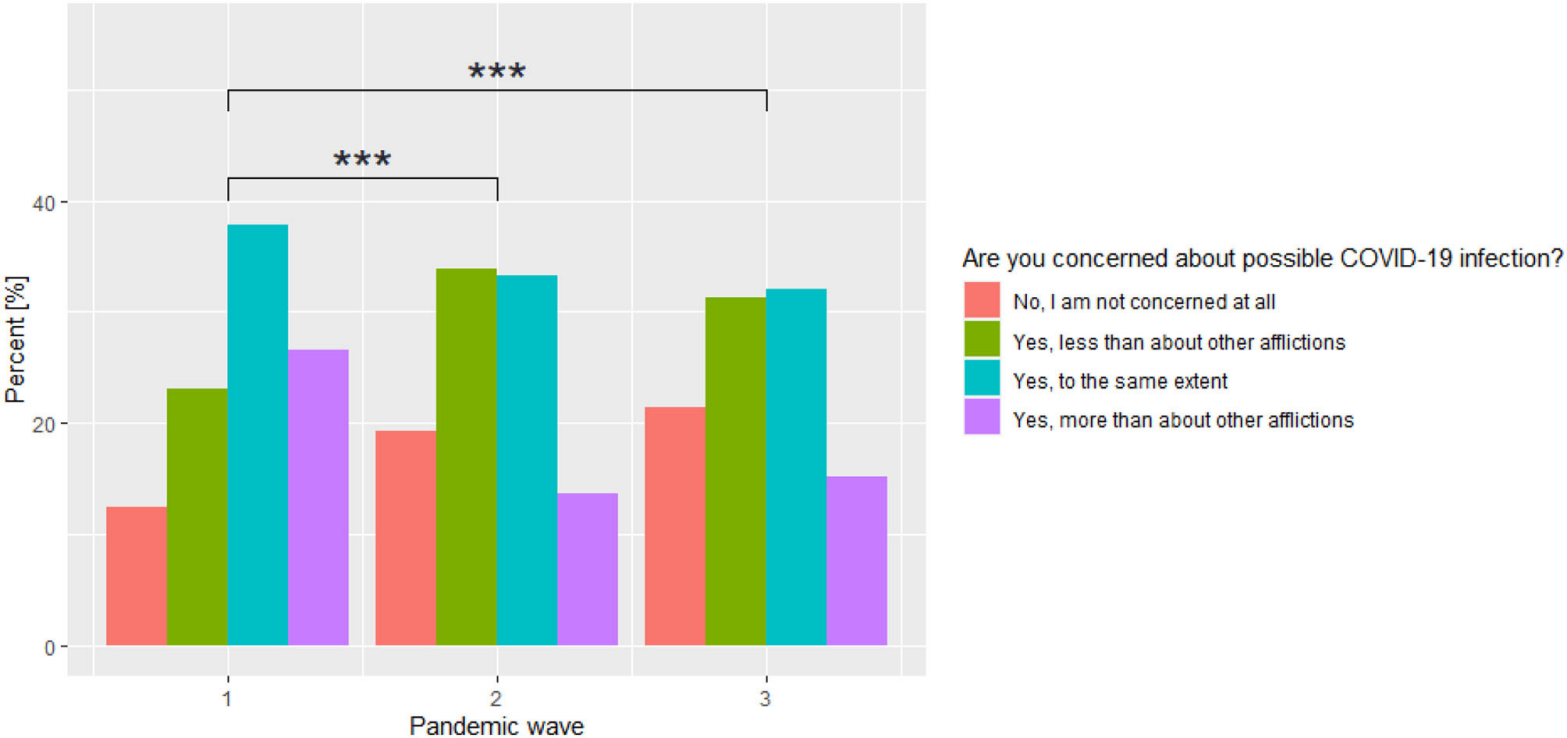
Comparison of the percentage of individuals who are concerned about COVID-19 infection across the three waves of the COVID-19 pandemic. ***p < 0.001.

The authors' own set of questions based on a 10-point Likert scale regarding anxiety about being infected with SARS-CoV-2 infection, as well as anxiety about neighbors in quarantine or neighbors being infected with COVID-19, were used for the assessment of the subjective sense of anxiety about contracting COVID-19 disease. The analysis of the subjective assessment of anxiety about contracting COVID-19 disease reveals a significantly statistical level of anxiety reduction as the COVID-19 pandemic continued (*p* < 0.0001). When comparing individual waves of the pandemic, the strongest anxiety reduction was observed between stage 1 and 2 of the study (*p* < 0.0001). That relationship was not observed when comparing stage 2 and 3 of the study. Similar relationships were observed when assessing the anxiety about neighbors in quarantine or neighbors being infected with COVID-19. There is a statistically significant difference between individual stages of the study (*p* < 0.0001) when assessing anxiety about contracting COVID-19 infection compared to other afflictions. Over time, the percentage of those who are concerned about SARS-CoV-2 infection more strongly than about other afflictions or to the same extent decreased, while the percentage of those who are not concerned about COVID-19 infection increased. The comparison of response rates across COVID-19 pandemic waves is shown in Figure 3. The assessment of the adherence to the Ministry of Health recommendations regarding COVID-19 prevention reveals its gradual reduction as the pandemic progressed. With each subsequent COVID-19 pandemic wave, the level of this adherence was significantly lower. A detailed comparison is summarized in Table 6.

**Table 6:** Comparison of mean scores of anxiety about being infected with SARS-CoV-2 and about neighbors in quarantine or neighbors being infected with COVID-19.

	**First wave**	**Second wave**	**Third wave**	***P*** ^*^
**Anxiety about being infected with COVID-19 disease**				
Mean	5.51	4.86	4.92	**<0.0001**
Comparison of individual COVID-19 pandemic waves			x	**<0.0001**
		x		**<0.0001**
	x			0.9799
**Anxiety about neighbors being infected with COVID-19**				
Mean	5.73	3.63	3.59	**<0.0001**
Comparison of individual COVID-19 pandemic waves			x	**<0.0001**
		x		**<0.0001**
	x			0.1829
**Anxiety about neighbors in quarantine**				
Mean	4.64	3.03	2.93	**<0.0001**
Comparison of individual COVID-19 pandemic waves			x	**<0.0001**
		x		**<0.0001**
	x			0.256
**Adherence to the Ministry of Health recommendations regarding SARS-CoV-2 prevention**				
Mean	8.67	7.63	7.10	**<0.0001**
Comparison of individual COVID-19 pandemic waves			x	**<0.0001**
		x		**<0.0001**
	x			**<0.0001**

* *Type-II ANOVA. The assessment of the adherence to the Ministry of Health recommendations regarding SARS-CoV-2 prevention. Significant effects (<0.05) are marked in bold*.

### Assessment of the Effect of Sociodemographic Factors on the Mean Scores of BDI, GAD-7, and MANSA

The effect of sociodemographic variables on the mean scores of BDI, GAD-7, and MANSA is summarized in detail in Table 7. There was a statistically significant relationship between the age of the respondent and the mean score of BDI and GAD-7—the higher the age, the lower the score of both scales. Men and individuals with a university degree obtained significantly lower scores on BDI and GAD-7 scales, with no difference in terms of pandemic waves. The reduction in income opportunities due to the pandemic significantly affected the final scores of each scale used. The BDI analysis revealed that the increase was significantly greater during the second wave of the COVID-19 pandemic (value 2.193; *SD* 0.766; *t* = 2.86; *p* = 0.004), similarly to the GAD-7 questionnaire (value 1.180; *SD* 0.469; *t* = 2.51; *p* = 0.012). It was observed that healthcare professionals had significantly lower BDI questionnaire scores and the score increased more slowly from wave to wave compared to non-healthcare workers (second wave: value −1.747; *SD* 0.887; *t* = −1.97; *p* = 0.049; third wave: value −2.182; *SD* 0.836; *t* = −2.61; *p* = 0.009).

**Table 7:** Effects of sociodemographic variables on scores of individual scales.

		**BDI**				**GAD-7**				**MANSA**			
		**Value**	***SD***	***t***	***p***	**Value**	***SD***	***t***	***p***	**Value**	***SD***	***t***	***p***
	Age	−0.097	0.018	−5.19	**0.000**	−0.035	0.011	−3.11	**0.001**	−0.002	0.024	−0.11	0.914
Sex	Male	−1.629	0.345	−4.72	**0.000**	−1.974	0.208	−9.48	**0.000**	−0.195	0.436	−0.45	0.653
Place of residence	Rural area	0.267	0.365	0.73	0.463	0.103	0.222	0.47	0.641	−0.315	0.461	−0.68	0.494
	Town of up to 50,000 inhabitants	0.332	0.393	0.84	0.398	−0.037	0.239	−0.16	0.875	−0.755	0.496	−1.52	0.128
	City of 50,000–250,000 inhabitants	−0.106	0.361	−0.29	0.769	−0.259	0.219	−1.18	0.238	0.294	0.456	0.65	0.518
Level of education	Higher (university degree)	−9.820	1.350	−7.27	**0.000**	−3.476	0.831	−4.18	**0.000**	8.717	1.720	5.07	**0.000**
	Incomplete higher	−6.949	1.355	−5.13	**0.000**	−2.861	0.834	−3.43	**0.000**	6.547	1.726	3.79	**0.000**
	Secondary	−6.320	1.362	−4.64	**0.000**	−2.605	0.838	−3.11	**0.001**	5.429	1.734	3.13	**0.001**
	Vocational	−4.363	1.895	−2.30	**0.021**	−1.657	1.167	−1.42	0.155	3.253	2.414	1.35	0.177
	Primary	0.636	2.763	0.23	0.817	−2.958	1.701	−1.74	0.082	6.887	3.519	1.96	0.050
Marital status	Married	−2.838	0.461	−6.15	**0.000**	−0.528	0.292	−1.81	0.070	3.015	0.602	5.01	**0.000**
	Partnership	−1.407	0.527	−2.67	**0.007**	−0.168	0.331	−0.51	0.610	2.869	0.683	4.20	**0.000**
Medical professionals	Yes	−1.939	0.464	−4.17	**0.000**	−0.037	0.283	−0.13	0.894	2.404	0.436	5.51	**0.000**
Earnings reduction	Yes	3.249	0.465	6.98	**0.000**	1.267	0.285	4.44	**0.000**	−4.757	0.586	−8.11	**0.000**
Psychiatric treatment	Yes	5.771	0.487	11.83	**0.000**	2.965	0.196	15.09	**0.000**	−5.819	0.409	−14.22	**0.000**

*Significant effects (<0.05) are marked in bold*.

### Correlations Between BDI, GAD-7, and MANSA Scores

Each stage of the pandemic reveals a positive correlation between GAD-7 and BDI (stage I: *r* = 0.7, *p* < 0.001; stage II: *r* = 0.73, *p* < 0.001; stage III: *r* = 0.75, *p* < 0.001). However, both GAD-7 and BDI reveal an inverse correlation compared to MANSA at each stage of the study (GAD-7: stage I: *r* = −0.51, *p* < 0.001; stage II: *r* = −0.59, *p* < 0.001; stage III: *r* = −0.632, *p* < 0.001; BDI: stage I*: r* = −0.63; *p* < 0.001; stage II: *r* = −0.712, *p* < 0.001; stage III: *r* = −0.74, *p* < 0.001).

## Discussion

The unanticipated pandemic outbreak in March 2020 changed the lives of many people in a significant way. Its dynamics and multifaceted nature have led some re-searchers to consider it a phenomenon of collective trauma (19). A pandemic state is associated with tremendous life instability, and it is characterized by an uneven course. This is due to the surge nature of infections and associated numerous restrictions imposed by the government to inhibit the transmission of the virus. When it comes to negative emotions recognized in society during the pandemic, such as sadness, fear and grief, uncertainty was prevalent emotion. This factor, resulting from a completely new stressor for Polish society—the pandemic situation, extremely negatively affects the human psyche (20). It is still impossible to assess the long-term social, and health impacts of the pandemic (6). Therefore, this study mainly aims to assess the mental state of the Polish people during the COVID-19 pandemic across its waves.

The study was conducted in three stages for each pandemic wave, respectively. Its results indicate a gradual increase in the frequency of depressive, and anxiety symptoms in the Polish population as the pandemic progressed. It should be noted that those changes were not uniform. Although restrictions regarding COVID-19 prevention were greatest, and longest during the third wave of the pandemic, there was a greater difference between the first and second wave than between the second, and third wave (14). On the one hand, it is obvious that due to the significant increase in infection and death rates, fear, and concern for one's own life arose in society. On the other hand, the slight difference between the second, and third wave points to a progressive partial adaptation to the pandemic situation (3). Psychological research implies that despite the much pandemic-related annoyance, some people observed also positive aspects of the pandemic state in their life, e.g., solidarity among local communities in support of the healthcare system, more leisure time, improved relationships with other people, increased sensitivity to their own mental, and physical health or hygiene (21).

Moreover, in the BDI analysis regardless of the stage of the survey, more than one third of the respondents obtained a score indicating the occurrence of at least mild depression. It should be noted that there were unidirectional changes in symptom severity, including an increase in the percentage of individuals whose scale score indicated the presence of depressive disorders, as well as a gradual increase in the percentage of individuals with moderate and severe depression as the pandemic progressed. Similar scores were found in the interpretation of GAD-7, where the number of people showing severe anxiety increased between the first, and third wave of the COVID-19 pandemic by nearly 5%. In addition to that, the observed anxiety, and depressive symptoms were significantly higher than those found in epidemiological studies conducted in Poland before the pandemic (22). An interesting relationship was found in a longitudinal study on the Spanish, and Chinese populations, where there was also an in-crease in depressive symptoms as the time of the restrictions regarding COVID-19 prevention prolonged. In contrast to the results of this study, anxiety symptoms remained on a high level since the beginning of the pandemic (23, 24). In a British longitudinal study, anxiety symptoms even decreased during successive stages of the pandemic despite persistently high levels of depressive symptoms and increased suicidal tendencies (9). This was justified by the fact that the unexpected global situation generated extremely strong anxiety, while deepening financial instability and social isolation had a greater impact on mood decline, which also seems to be reflected in this study (23).

Furthermore, it was also found that as the pandemic progressed, the respondents had significantly lower scores for anxiety about their own or their neighbors' possible COVID-19 infection, as well as they revealed less rigorous adherence to the Minister of Health's recommendations to reduce the virus transmission. The longitudinal study on Chinese, and American populations also revealed a gradual decrease in anxiety about virus infection. This was thought to be related to the fact that mortality turned out to be lower than initially anticipated and safeguards were implemented to reduce the risk of virus transmission (25). On the contrary, the population of Israel revealed greater willingness to adhere to public health recommendations as the pandemic progressed (26). The trend present in the Polish population may be due to low trust of the Polish people in media coverage of the pandemic situation, and also due to the fact that be-tween the second, and third wave of the COVID-19 pandemic, a vaccination programme was implemented in Poland, which gave some people a greater sense of security (27).

At the same time, there was no unidirectional shift in quality-of-life scores on the MANSA scale. According to the European Foundation for the Improvement of Living, and Working Conditions, the EU inhabitants assessed their quality of life significantly lower only during the third wave of the COVID-19 pandemic as their economic situation worsened (28). In a German study of families, the statistically significant deterioration in quality of life was obtained as early as the second wave of the pandemic, and the strength of the effect was dependent on the quality of family relationships (29). Quality of life is undoubtedly a complex and difficult parameter to assess, which is affected by many factors. Some of the variables that could affect the outcome of the assessment improved (e.g., anxiety about developing COVID-19 disease) and others, such as economic situation, worsened, which may explain the balanced results obtained in subsequent stages of this study. The correlation analysis between the scales clearly reveals a decrease in the quality-of-life assessment as depressive and anxiety symptoms increase. Moreover, unlike BDI and GAD-7, age, and sex did not differentiate MANSA scores.

When analyzing the collected data, the significant influence of socio-economic parameters on the scores obtained by the respondents should be noted. Significantly worse mental health status was obtained by women and young people on all three scales. According to research reports, young age and female sex increase the risk of increasing depressive and anxiety symptoms. This may be due to older people's greater mental resilience, greater life experience, habitual solitude, and better emotion regulation (30, 31). Singles, individuals with prior psychiatric treatment and those whose in-come opportunities were reduced during the pandemic showed a similar negative trend. Based on previous economic crises, it was observed that job loss, higher work-loads or pay reductions increase the frequency of depressive and anxiety disorders and suicides (32, 33). In another study conducted during the pandemic, it was found that ruminating and worrying accompanying loneliness had a greater effect on depressive symptoms than anxiety symptoms, which is also observed in this study when taking into consideration the strength of the effect (34). At the same time, numerous studies have revealed that individuals with mental illness, compared to the general population, showed increased susceptibility to stress during a crisis already before the pandemic and they frequently had exacerbated psychopathology (35).

The scores indicating lower intensity of psychological problems were obtained by healthcare professionals; however, in another Polish study using GHQ-28, medical professionals working in direct exposure to COVID-19 obtained higher scores on this scale compared to the general population. Therefore, it should be emphasized that this is a heterogeneous group and the obtained scores may differ significantly in terms of individual occupational subgroups (36). This has also been confirmed in many other world studies (37, 38). Economic stability seems to be an important element for medical professionals. In the era of the pandemic, healthcare professionals were not exposed, like other professions, to reduction or even complete freezing of earnings due to government restrictions—as known from previous reports, economic stability is one of the strongest predictors of psychological well-being (32, 33). Moreover, as the pandemic continued, working conditions improved, access to personal protective equipment in-creased and the management of a person suspected or infected with SARS-CoV-2 was more clearly defined. Furthermore, individuals with a university degree were more resilient to depressive and anxiety disorders, and a similar pattern was also found in a study concerning the Chinese population (39). On the other hand, as a pandemic continues, and hence a significant workload, the mental condition of medical workers may deteriorate. According to WHO, the condition of medical workers is an important aspect of the fight against the COVID-19 pandemic and the need for support for psychiatric care is high (40, 41). Therefore, it is necessary to implement appropriate psychological support strategies as well as to ensure safe working conditions in order to maintain the psychological comfort of employees (38, 42–44). Failure to do so may lead to a deterioration of mental health, which may result in a reduction in the quality of services provided, and even professional burnout.

The authors are aware of the strengths and weaknesses of this study. According to the available data, this is one of the first cross-sectional studies concerning the psychological well-being of the Polish people that includes data obtained from all three waves of the COVID-19 pandemic, which demonstrates its strength and innovation. However, the limitation of this review is undoubtedly the lack of representativeness of the study group with respect to Polish society. The overwhelming predominance of women and the low mean age of respondents may influence the final result of the observation. Another methodological limitation is the data collection method in the form of an anonymous survey distributed through a social networking site. As a result, the authors have no way of verifying the number of people who started but did not complete the survey or the number of people who knew about the survey. On the other hand, due to the prevailing sanitary and epidemiological restrictions, that was the only way to safely conduct a study on this scale. Furthermore, non-lockdown periods were not taken into consideration although this might have contributed to obtaining more robust conclusions, as longitudinal studies from other countries revealed gradual improvements in psychological well-being as prevailing restrictions regarding COVID-19 prevention were loosened. Due to the nature of the study (full anonymity and the way the questionnaire was distributed), the authors of this report could not provide psychological support to respondents. One can hope that participation in this study prompted the participants to take a closer look at their own mental health and, if necessary, seek medical assistance.

The authors intend to continue to conduct observations, and the obtained results will provide a more precise way to determine progressive changes in the severity of mental disorders in the population, which will help better understand the complexity of the impact of the pandemic on mental health. Another study is also necessary due to the likely fourth pandemic wave associated with the Delta variant of coronavirus (45). To this end, it would be worthwhile to consider extending the authors' own questionnaire to include newly developed tools designed for assessing psychological well-being in relation to the ongoing COVID-19 pandemic, e.g., the Coronavirus Anxiety Scale (46).

In summary, the COVID-19 pandemic is an unexpected and unique experience. Its numerous implications affect people's mental health. Therefore, there is a need for constant monitoring of this phenomenon and searching for systemic solutions that can significantly reduce the destructive impact of the pandemic on mental health. The example of such a solution could be the use of workplaces and schools for providing training in mental health hygiene, including stress management techniques (47).

## Conclusions

The COVID-19 pandemic has a significant impact on the mental health of the Polish people. This effect is not uniform and the severity of depressive and anxiety symptoms varies from wave to wave. As the pandemic continues, there is a unidirectional shift toward increased anxiety and depressive disorders. The impact of the COVID-19 pandemic on a subjective sense of quality of life is not uniform, with particular components worsening and others improving as the pandemic continues. Women, younger people, singles, and those treated psychiatrically in the past have significantly more severe psychotic symptoms. There is a need to continue to monitor the impact of the ongoing global epidemic situation on mental well-being to assess the long-term effects of the pandemic on mental health.

## Data Availability Statement

The raw data supporting the conclusions of this article will be made available by the authors, without undue reservation.

## Ethics Statement

The studies involving human participants were reviewed and approved by Bioethics Committee of the Wroclaw Medical University. The Ethics Committee waived the requirement of written informed consent for participation.

## Author Contributions

MB, KK, BB, and AM-M: conceptualization, methodology, writing—original draft, and writing—review and editing. KK: formal analysis. MB: funding acquisition and visualization. MB, BB, and AM-M: investigation and supervision. All authors contributed to the article and approved the submitted version.

## Funding

This research was founded by the Wroclaw Medical University SUB.C290.21.010.

## Conflict of Interest

The authors declare that the research was conducted in the absence of any commercial or financial relationships that could be construed as a potential conflict of interest.

## Publisher's Note

All claims expressed in this article are solely those of the authors and do not necessarily represent those of their affiliated organizations, or those of the publisher, the editors and the reviewers. Any product that may be evaluated in this article, or claim that may be made by its manufacturer, is not guaranteed or endorsed by the publisher.
