# Supplementary material for: Impact of the COVID-19 Pandemic on Mental Well-Being. A Nationwide Online Survey Covering Three Pandemic Waves in Poland
Source: Front Psychiatry. 2021 Dec 17;12:804123. doi: 10.3389/fpsyt.2021.804123 (PMC8718800; doi:10.3389/fpsyt.2021.804123)
Supplement: Supplementary file 1 [file Table_1.pdf]

Supplementary Material

Table S1 . The level of satisfaction (including three of seven answers which are positive: could not be better/ I am satisfied/ I am somewhat satisfied) for the individual questions of the MANSA scale by survey stage.

Question	Stage 1 (n=2467) N (%) M (SD)*	Stage 2 (n=1626) N (%) M (SD)*	Stage 3 (n=1696) N (%) M (SD)*	χ2	Effect size****	p
Mean score*	60.62 ±12.73	61.54 ±12.61	60,18 ±13,07	---	0.002	0.007**
1. How satisfied are you with your life as a whole today?	1379 (56.1)	856 (52.6)	855 (50.4)	14.14	0.049	0.001***
2. How satisfied are you with your job (other professional activities or schooling)?	1323 (53.8)	786 (48.3)	795 (46.9)	21.97	0.062	<0.001***
3. How satisfied are you with your financial situation?	1298 (52.8)	836 (51.4)	789 (46.5)	16.01	0.053	<0.001***
4. How satisfied are you with the number and quality of your friendships?	1362 (55.4)	956 (58.8)	948 (55.9)	5.19	0.030	0.075***
5. How satisfied are you with your leisure activities (hobbies)?	1165 (47.4)	723 (44.5)	685 (40.4)	20.15	0.059	<0.001***
6. How satisfied are you with your accomodation?	1583 (64.4)	1097 (67.5)	1067 (62.9)	7.96	0.037	0.019***

7. How satisfied are you with your personal safety?	1555 (63.3)	1157 (71.2)	1138 (67.1)	26.47	0.068	<0.001***
8. How satisfied are you with the people that you live with?	1734 (70.6)	1198 (73.7)	1214 (71.6)	4.41	0.028	0.110***
9. How satisfied are you with your sex life?	1102 (44.9)	757 (46.6)	777 (45.8)	1.02	0.013	0.600***
12. How satisfied are you with your relationship with your family?	1689 (68.7)	1097 (67.5)	1118 (65.9)	3.35	0.024	0.187***
13. How satisfied are you with your physical health?	1284 (52.3)	766 (47.1)	775 (45.7)	18.61	0.057	<0.001***
12. How satisfied are with your mental health?	1183 (48.1)	656 (40.3)	640 (37.7)	51.13	0.094	<0.001***

** Kruskall Wallis test

*** $\chi 2$ Pearson test

**** $\eta 2$ for Kruskall Wallis test and Fi / Cramer's V for $\chi 2$ Pearson test
